# Viral prevalence, clinical profiles and comparison of severity scores for predicting the mortality of adults with severe acute respiratory infections

**DOI:** 10.3906/sag-1807-231

**Published:** 2019-06-18

**Authors:** Hakan AYDIN, Halil DOĞAN, Doğaç Niyazi ÖZÜÇELİK, Mehmet KOÇAK, Osman Avşar GÜL

**Affiliations:** 1 Department of Emergency Medicine, Bakırköy Dr. Sadi Konuk Training and Research Hospital, University of Health Sciences, İstanbul Turkey

**Keywords:** Severe acute respiratory infection, severity scores, influenza viruses

## Abstract

**Background/aim:**

The aim of this study was to determine the accuracy of severity scores for predicting the 28-day mortality among adults with severe acute respiratory infection (SARI) admitted to the emergency department.

**Materials and methods:**

This study included 159 consecutive adult patients with SARI admitted to the emergency department of a tertiary hospital. A standard form was filled out in order to record demographic information, clinical parameters, laboratory tests, and radiographic findings of the patients. CURB-65, PSI, SIRS, qSOFA, SOFA and APACHE II scores were compared between the survivor and nonsurvivor groups.

**Results:**

Of 159 patients included in the study, 38.4% were positive for respiratory viruses and 28.3% were positive for influenza viruses. 35.8% of the patients were admitted to an intensive care unit (ICU) and the mortality rate was 36.5%. The area under the receiver operating characteristic curve of CURB-65, PSI, SIRS criteria, qSOFA, SOFA and APACHE II scores were 0.717, 0.712, 0.607, 0.683, 0.755, and 0.748, respectively in predicting mortality and 0.759, 0.744, 0.583, 0.728, 0.741, and 0.731, respectively in predicting ICU admission.

**Conclusion:**

SOFA and APACHE II were more accurate than SIRS in predicting the 28-day mortality among adults with SARI. There was no significant difference among these scores in terms of other multivariate comparisons.

## 1. Introduction

Acute respiratory infection (ARI) is a major cause of morbidity and mortality worldwide and tends to be a rapidly progressive illness due to pathogens having the potential for large scale epidemics. According to the World Health Organization (WHO), these annual epidemics result in 3–5 million severe illness cases and 290–650 thousand deaths all around the world. Influenza-like illnesses (ILI), a subset of ARIs, accounted for approximately 1.9 million deaths in children below 5 years of age worldwide in 2000 [1]. In addition to the ILI outpatient surveillance, the WHO recommended the member states to start a monitoring for severe ARIs (SARIs) in hospitalized patients after the 2009 influenza pandemic [2].

Severe acute respiratory infection (SARI) is one of the leading causes of sepsis among adults. Identifying patients with SARI and sepsis having higher risk of mortality is crucial to anticipate prognosis and follow treatment program. Many severity-scoring systems have been developed to assess the severity of these patients. Current guidelines suggest the use of various severity scores such as CURB-65 and pneumonia severity index (PSI) in order to classify patients with community-acquired pneumonia (CAP) [3]. In 2016, the third international consensus definitions for sepsis and septic shock proposed a new definition and a scoring system based on sequential (sepsis-related) organ failure assessment (SOFA) score instead of systemic inflammatory response (SIRS) criterion [4], which has been used to define sepsis for a long time [5]. In addition, the Sepsis-3 task force proposed the quick SOFA (qSOFA) as a simpler scoring system for the initial screening of patients at high risk for sepsis [5,6]. However, debates about the performances of existing classifications are still ongoing.

This study was conducted to evaluate and compare the accuracy of CURB-65, PSI, SIRS criteria, qSOFA, SOFA, and APACHE II for predicting mortality among adults with SARI.

## 2. Materials and methods

### 2.1. Design, setting, and patients

This prospective, observational, single-center, and cross-sectional study included consecutive patients (≥ 18 years of age) who were admitted to the emergency department (ED) of Bakırköy Dr. Sadi Konuk training and research hospital (BEAH) in İstanbul, Turkey between January 2016 and March 2016 and hospitalized due to the diagnosis of SARI. The hospital has more than 612 beds and the number of ED patient visits is approximately 230,000 per year. Patients who did not meet the criteria of SARI case definition, had incomplete information, and lacked the results of nasal swab tests were excluded from the study.

Before conducting the study in accordance with the principles of Declaration of Helsinki, approval of the local ethics committee (reference number: 2016.01.33) was obtained. Written informed consent was obtained from all patients or their representatives.

### 2.2. Definitions

In the present study, SARI case was defined as an acute respiratory infection with a history of fever or measured fever of ≥ 38 ºC and cough, developing within the last ten days and requiring hospitalization [2]. ILI case was defined as an acute respiratory infection with a measured fever of ≥ 38ºC and cough developing within the last ten days [2].

### 2.3. Data collection

The following data were collected in the ED: age, sex, presence of individuals with similar symptoms in the circle, presence of pregnancy, trimester of pregnancy, vaccination status, onset of the complaint, chronic diseases, and smoking status. Hemodynamic parameters, blood pressure, pulse rate, respiration rate, pulse oximeter, oxygen saturation, body temperature, and consciousness status were also evaluated. Since the definition of altered consciousness was not equivalent to a Glasgow Coma Scale (GCS) score of < 15, the presence of altered mental status was recorded independent of GCS and was clinically determined by a physician. Moreover, the findings of physical examination and laboratory tests (complete blood cell count, biochemical parameters, and blood gas), radiographic findings, and clinical outcomes (intubation, ICU admission) were recorded and the nasal swabs were obtained. The scores of CURB-65, PSI, SIRS criteria, qSOFA, SOFA and APACHE II were calculated for all patients admitted to the ED and those who were admitted to the ED more than once. Their first laboratory results and their data collected after the first visit were included in the study as long as the inclusion criteria were fulfilled.

### 2.4. Study protocol and follow-up evaluation

All patients included in the study were treated and followed up according to the Adult Infectious Diseases Society of America/American Thoracic Society consensus guidelines of the CAP in adults [3]. Evaluation of the admitted patients and hospitalization decision were based on patients’ medical conditions and vital parameters including CURB-65 and PSI scores and performed by a specialist of the department of emergency medicine or the department of infectious diseases and clinical microbiology. The primary endpoint was death. The patients were divided into two groups as survivor and nonsurvivor groups based on survival rates on a 28-day scoring scale. Additionally, the patients who died after the 28th day during their treatment in the hospital or ICU were included in the nonsurvivor group. The secondary outcomes were the admission to the ICU and length of hospital stay. Information regarding survival or death of the patients was obtained from the patients or their relatives by phone calls within 28 days. The data were further confirmed by reviewing the medical records of the hospital. For deaths outside the hospital, the national death notification system, reporting all deaths in the study area on a daily basis, was sought.

### 2.5. Statistical analysis

Demographic, clinical, and laboratory variables were compared between the survivor and nonsurvivor groups. Normality of the data was tested using the Kolmogorov–Smirnov test. Differences in continuous variables were assessed using the Mann–Whitney U test and categorical variables were compared using the Chi-square test or Fisher’s exact test. Categorical variables were expressed as absolute values and percentages. Continuous variables were expressed as mean ± standard deviation for normally distributed data or medians within 25th to 75th interquartile range (IQR) for nonnormally distributed data. All analyses were performed using the SPSS statistics version 24.0 (IBM Corp, Armonk, NY, USA). Receiver operating characteristics (ROC) analysis was carried out and the area under the curve (AUC) was compared using MedCalc statistical software version 17.9.7 (Medcalc Software bvba, Ostend, Belgium) to evaluate the predictive performances of CURB-65, PSI, SIRS, qSOFA, SOFA, and APACHE II scores. Sensitivity, specificity, positive predictive value (PPV), and negative predictive value (NPV) were also calculated based on the cut-off point. For all tests, a P value of < 0.05 was accepted as statistically significant.

## 3. Results

**Figure 1:**
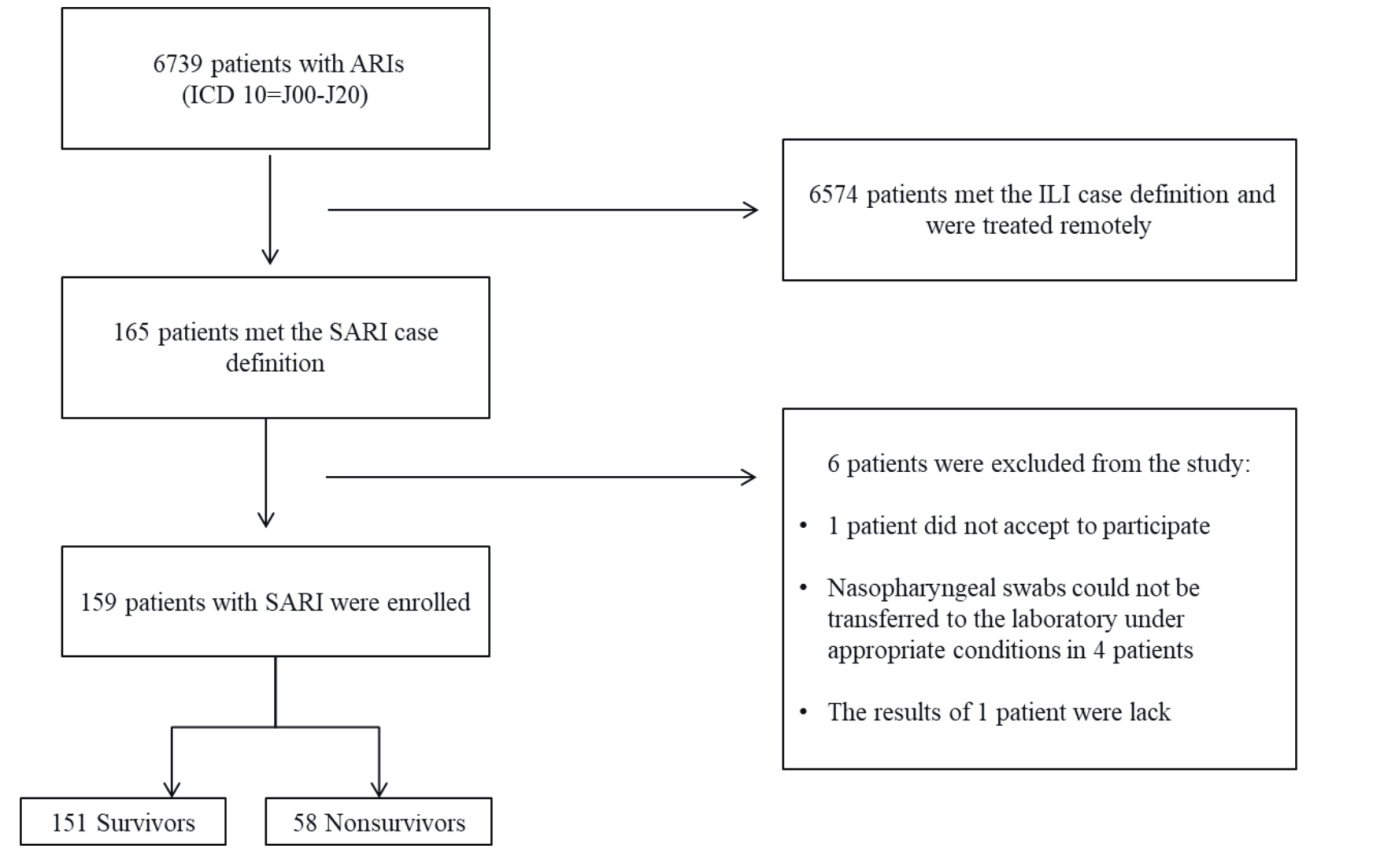
Patient flowchart.

Among all patients admitted to the ED of BEAH between January 05, 2016 and March 31, 2016, 6,739 patients were evaluated as ARI (International classification of diseases tenth revision (ICD 10) = J00-J20). 6,574 of these patients who met the ILI case definition were treated remotely and 165 were hospitalized based on the SARI case definition. Nasopharyngeal swabs could not be transferred to the laboratory under appropriate conditions in 4 of the hospitalized patients, the results of 1 patient were lack, and 1 patient did not accept to participate in the study. After excluding these patients, 159 hospitalized patients with SARI case definition were enrolled in the present study (Figure 1).

The median age of the 159 patients (females, 45.9%) was 66 years (IQR, 48.5-81 years). Forty-four patients (22.7%) were aged between 18 and 49 years and 86 (54.5%) were above 65 years of age. Of the patients, 6.1% were the residents out of the surveillance area, 3.1% traveled abroad within the last 10 days, and 58.5% had individuals with similar symptoms in their circle. Of the patients, 79.2% had one or more comorbid diseases. The most common comorbid disease was found to be hypertension at the rate of 40.9%. Of the patients, 35.2% were active smokers, only 2 patients were pregnant, and 30.8% were those in need of nursing care. The rate of admission to the ED was observed to be the highest on the 3rd day of the disease with 28.30% (n = 45). Of the survival patients, 30.7% were treated within 72 h or less, 28.7% were treated in 3 to 7 days, and 39.6% were treated within 8-28 days. Among patients with SARI, 35.8% were admitted to ICU and 36.5% died. The median survival time of the patients who died during the follow-up period was 6.5 days. Accordingly, it was found that there was a significant difference between the survivor and nonsurvivor patients in terms of heart rate, respiratory rate, and oxygen saturation (P = 0.015, P < 0.001, and P < 0.001, respectively). In addition, altered mental status (P < 0.001), respiratory distress (P = 0.006), current smokers (P = 0.023), and a history of diabetes mellitus (P < 0.001) or oncologic diseases (P = 0.008) were associated with high mortality. Comparison of the results of the survived and nonsurvived patients in terms of baseline characteristics and secondary outcomes are presented in Table 1.

**Table 1:** Baseline characteristics and secondary outcomes in the whole study patients and survived and nonsurvived groups.

	Overall (n = 159)n (%), median (IQR)*	Survivor (n = 101)n (%), median (IQR)*	Nonsurvivor (n = 58)n (%), median (IQR)*	P
Demographic data				
Age, years	66 [48.5–81]	66 [48–78]	66.5 [52–83]	0.342
Female	73 (45.9)	48 (47.5)	25 (43.1)	0.590
Current smoker	56 (35.2)	29 (28.7)	27 (46.6)	0.023
Nursing home resident	49 (30.8)	28 (27.7)	21 (36.2)	0.265
Oseltamivir treatment	79 (49.7)	55 (54.5)	24 (41.4)	0.112
Symptoms and signs				
Fever (T ≥ 38ºC)	94 (59.1)	60 (59.4)	34 (58.6)	0.923
Respiratory distress	142 (89.3)	85 (84.2)	57 (98.3)	0.006
Altered mental status	49 (30.8)	20 (19.8)	29 (50.0)	<0.001
Medical history				
Chronic heart disease	46 (28.9)	29 (28.7)	17 (29.3)	0.936
Chronic pulmonary disease	48 (30,2)	33 (32,6)	15 (25,9)	0.269
Diabetes Mellitus	35 (22.0)	10 (9.9)	25 (43.1)	<0.001
Hypertension	65 (40.9)	43 (42.6)	22 (37.9)	0.566
Oncologic diseases	25 (15.7)	10 (9.9)	15 (25.9)	0.008
Chronic neurological disease	32 (20.1)	20 (19.8)	12 (20.7)	0.893
Hemodynamic parameters				
Forehead temperature, ºC	38.0 [37.7–38.2]	38.0 [37.8–38.2]	38.0 [37.6–38.2]	0.649
Systolic blood pressure, mmHg	110 [93.5–123]	110 [95–122]	109 [89–127]	0.577
Diastolic blood pressure, mmHg	66 [57–78]	67 [58–78]	61 [56–78]	0.261
Heart rate, beats/min	106 [95–117]	104 [94–114]	112.5 [96–124]	0.015
Respiratory rate, breaths/min	24 [21.5–28]	22 [20–26]	26 [24–30]	<0.001
Oxygen saturation (%)	86 [78–88.5]	88 [80–89]	81.7 [74–86]	<0.001
Secondary outcomes				
LOS in hospital or death. Day	6 [2–13.5]	6 [2–11]	6.5 [2–20]	0.277
Intensive care unit	72 (35.8)	19 (26.4)	53 (73.6)	<0.001

*Continuous variables were expressed medians with 25th to 75th interquartile range (IQR), LOS: length of stay.

Radiographic examination of the patients revealed that bilateral involvement (P = 0.001) and multilobar involvement (P = 0.002) were significantly higher in the nonsurvived group than in the survived group (Table 2).

**Table 2:** Laboratory results and radiographic findings in the whole study patients and survived and nonsurvived group.

	Overall (n = 159)	Survivor (n = 101)	Nonsurvivor (n = 58)	P
Laboratory results, median (IQR)1				
WBC (103/μL)	10.7 [7.01–15.3]	10.2 [7.26–14.1]	11.65 [6.82–18.4]	0.202
Neutrophils	8.48 [5.33–12.3]	7.6 [5.04–11.0]	8.96 [6.07–15.0]	0.092
Hematocrit	36.8 [31.8–40.8]	37.7 [32.9–41.9]	35.1 [29.9–39.2]	0.013
C-reactive protein (mg/dL)	7.55 [3.05–16.2]	6.39 [2.65–14.3]	9.46 [3.24–22.8]	0.116
Urea. mg/dL	48.0 [31–84]	45.0 [28–66]	61.5 [39–107]	<0.001
Creatinine. mg/dL	1.04 [0.76–1.47]	1.02 [0.75–1.33)	1.14 [0.80–2.14]	0.092
Sodium, mM	136 [133.5–140.5]	137 [134–140]	134 [129–142]	0.112
Calcium, mM	8.52 [8.0–8.96]	8.64 [8.28–9.01]	8.31 [7.64–8.77]	0.005
Albumin	3.6 [3.1–4.0]	3.8 [3.3–4.1]	3.35 [2.7–3.7]	<0.001
PaO2. mmHg	54 [47.9–58.0]	55.4 [50.0–59.0]	53.0 [44.6–56.3]	0.007
Lactate (mg/dL)	1.80 [1.21–2.79]	1.6 [1.19–2.42]	2.25 [1.27–3.60]	0.009
Radiographic findings, N (%)2				
Infiltration	156 (98.1)	99 (98.0)	57 (98.3)	0.909
Bilateral lung involvement	102 (64.2)	55 (54.5)	47 (81.0)	0.001
Multilobar involvement	108 (67.9)	60 (59.4)	48 (82.8)	0.002
Pleural effusion	60 (37.3)	35 (34.7)	25 (43.1)	0.290

1Continuous variables were expressed medians with 25th to 75th interquartile range (IQR). 2Categorical variables were expressed as absolute values and percentages. INR: international normalized ratio, PaO2: arterial partial pressure of oxygen, WBC: white blood cell.

Of 159 patients admitted to the ED, 78 (18%) fulfilled acute respiratory distress syndrome (ARDS) criteria. The mortality rate in patients with ARDS was 67.9% and significantly increased (P < 0.001).

### 3.1. Etiologies

According to the RT-PCR results, at least one respiratory virus was detected in 38.4% (n = 61) of the patients; whereas, influenza viruses were detected in 28.3% (n = 45) of the patients. Table 3 shows the distribution of the respiratory viruses among the patients.

**Table 3:** Distribution of the respiratory viruses among the study patients (n=159).

Identified respiratory viruses	All patients n (%)	Survivors n (%)	Nonsurvivors n (%)	P
Inﬂuenza viruses	45 (28.3)	31 (30.7)	14 (24.1)	0.377
Influenza A	42 (26.4)	29 (28.7)	13 (22.4)	0.396
H1N1pdm09	24 (15.1)	15 (14.9)	9 (15.5)	0.910
H3N2	18 (11.3)	14 (13.39)	4 (6.9)	0.194
Influenza B	3 (1.9)	3 (1.9)	1 (1.7)	0.984
Non-influenza respiratory viruses	18 (11.3)	9 (8.9)	9 (15.5)	
Respiratory syncytial virus	6 (3.8)	3 (3.0)	3 (5.2)	0.179
Coronavirus	7 (4.4)	2 (2.0)	5 (8.6)	0.029
Human metapneumovirus	4 (2.5)	3 (30)	1 (1.7)	0.681
Rhinoviruses	1 (0.6)	1 (1)	0	
At least one virus detected	61 (38.4)	40 (39.6)	21 (36.2)	0.672
No virus identified	98 (61.6)	98 (61.6)	37 (63.8)	
Total	159 (100)	151 (100)	58 (100)	

Twenty-nine (64.4%) of the SARI patients with influenza virus received oseltamivir. While 17 (58.6%) of these 29 patients received it on the day of admission; 21 (72.4%) patients received it within 48 hours after illness onset. The mortality rate was 27.6% in the patients receiving oseltamivir and 37.6% in those not receiving (P = 0.492). The mortality rate was 15.4% in the oseltamivir treatment within first 24 h after admission to the emergency department and 32.3% in the oseltamivir treatment within the first 48 h after the onset of symptoms (P = 0.461).

Of the 159 SARI cases, 69 (43%) patients had specimens obtained for blood culture and 29 (43%) were blood culture positive for 32 bacterial and candida pathogens: 14 (20.2%) of these 69 patients were positive for methicillin-resistant staphylococcus aureus (MRSA), 2 (2.8%) for methicillin-susceptible staphylococcus aureus, 4 (5.6%) for candida albicans, 3 (4.3%) for klebsiella pneumoniae, 4 (5.7%) for streptococcus pneumoniae, 3 (4.3%) for staphylococcus hominis, and 2 (2.8%) for enterobacter species. Twenty-eight (15.7%) patients had specimens obtained for endotracheal aspirate (eta) culture and 20 (71.4%) had eta culture positive for 22 bacterial and candida pathogens: 8 (28.5%) of the 28 patients were positive for acinetobacter species, 6 (21.4%) for candida albicans, 2 (0.7%) for candida species, 2 (0.7%) for klebsiella pneumoniae, 2 (0.7%) for stenotrophomonas maltophilia, and 2 (0.7%) for MRSA. Specimens were obtained from thirty (18.8%) patients for sputum culture, and 8 (44.4%) of them were sputum culture positive: 6 (2%) were positive for escherichia coli and 2 (0.6%) were positive for MRSA.

Etiologically, the presence of virus (P = 0.672) or bacteria (P = 0.109) alone was not effective in terms of mortality; however, the mortality rate increased in patients for whom both virus and bacteria were observed. Blood cultures were performed in 18% (n = 11) of the patients with SARI who were diagnosed with a virus and 54.5% (n = 6) of these patients had growth in their blood cultures. While all patients with growth in their blood culture died, all patients without growth in their blood culture were discharged (P = 0.002).

All patients received initial empiric antibiotic therapy. Most frequent regimens were beta-lactam plus macrolides (n = 79, 49.6%), beta-lactam plus fluoroquinolones (n = 66, 41.5%), and beta-lactam plus linezolid (n = 18, 6.2%). 

### 3.2. Comparison of severity scores

All six scoring systems can stratify according to risk of the 28-day mortality, even though none of them are particularly accurate. It was observed that the mortality rate was lower in the patients with low scores (5.8% for those with a CURB-65 score of 0, 0% for those with a PSI score of 1 and 2, 13.6% for those with a SIRS criteria score of 1, 15.3% for those with a qSOFA score of 0, 5.0% for those with a SOFA score of 1, and 15.1% for those with an APACHE II score of ≤ 9 (Table 4)). Moreover, the mortality rate was observed to be higher in the patients with high scores (58.6% for those with a PSI stage of 5, 54.5% for those with a qSOFA score of 3, 53.3% for those with a SIRS criteria score of 4, 84.2% for those with an APACHE II score of ≥ 25, 100% for those with a CURB-65 score of 5, and SOFA score of ≥ 7 (Table 4)). 

**Table 4:** Distribution of patients according to the severity scores.

	Score	Survivors, n (%)	Nonsurvivors, n (%)
CURB-65	0	16 (15.8)	1(1.7)
	1	32 (31.7)	11(19)
	2	34 (33.7)	18 (31)
	3	17 (16.8)	14 (24.1)
	4	2 (1.9)	9 (15.5)
	5	0	5 (8.6)
PSI	1	6 (5.9)	0
	2	9 (8.9)	0
	3	28 (27.7)	7 (12.1)
	4	39 (38.6)	24 (41.3)
	5	19 (18.8)	27 (46.6)
SIRS	0	2 (1.9)	0
	1	19 (18.8)	3 (5.1)
	2	28 (27.7)	17 (29.3)
	3	45 (44.6)	30 (51.7)
	4	7 (6.9)	8 (13.8)
qSOFA	0	22 (21.8)	4 (6.9)
	1	50 (49.5)	18 (31)
	2	24 (23.8)	30 (51.8)
	3	5 (4.9)	6 (10.3)
SOFA	1	19 (18.9)	1 (1.7)
	2	33 (32.7)	11 (19)
	3	18 (17.8)	9 (15.5)
	4	18 (17.8)	9 (15.5)
	5	11(10.9)	8 (13.8)
	6	2 (1.9)	4 (6.9)
	> 7	0	16 (27.5)
APACHE II	0–9	28 (27.7)	5 (8.6)
	9–14	37 (36.6)	10 (17.2)
	15–19	21 (20.8)	14 (24.1)
	20–24	12 (11.9)	13 (22.4)
	≥ 25	3 (3.0)	16 (27.6)

APACHE II: Acute physiology and chronic health evaluation 2, CURB65: confusion, urea > 7 mmol/L, respiratory rate 30/min, low systolic (< 90 mm Hg) or diastolic blood pressure (≤ 60 mm Hg), and age 65 years, PSI: pneumonia severity index, SIRS: systemic inflammatory response syndrome; SOFA: sequential (sepsis-related) organ failure assessment, qSOFA: quick SOFA.

**Figure 2:**
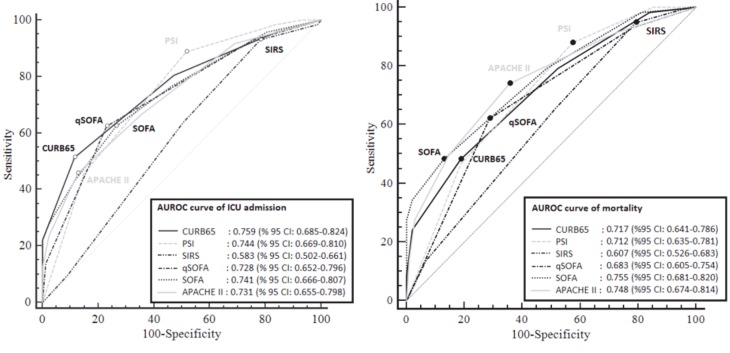
Area under the receiver operating characteristic (AUROC) curve. APACHE II: acute physiology and chronic health evaluation 2, CURB65: confusion, urea >7 mmol/l, respiratory rate 30/min, low systolic (< 90 mmHg) or diastolic blood pressure (≤ 60 mmHg) and age 65 years, PSI: pneumonia severity index; SIRS: systemic inflammatory response syndrome, SOFA: sequential (sepsis-related) organ failure assessment, qSOFA: quick SOFA scores for predicting intensive care unit (ICU) admission and for predicting mortality in patients with severe acute respiratory infection (SARI).

Discrimination of the 28-day mortality was also significantly higher in SOFA (AUC 0.755, 95% CI 0.681–0.820) or APACHE II (AUC 0.748, 95% CI 0.674–0.814) scores compared to SIRS criteria (AUC 0.607, 95% CI 0.526-0.683, P = 0.01 for both comparisons) (Figure 2). There was no significant difference in multiple pairwise comparisons between CURB-65 and PSI (P = 0.89), CURB-65 and SIRS (P = 0.06), CURB-65 and qSOFA (P = 0.49), CURB-65 and SOFA (P = 0.34), CURB-65 and APACHE II (P = 0.43), PSI and SIRS (P = 0.07), PSI and qSOFA (P = 0.58), PSI and SOFA (P = 0.32), PSI and APACHE II (P = 0.30), SIRS and qSOFA (P = 0.14), qSOFA and SOFA (P = 0.14), qSOFA and APACHE II (P = 0.18), SOFA and APACHE II (P = 0.86). For predicting the 28-day mortality among all patients with SARI, SOFA > 4 was the most specific scoring system (87.13%, 95% CI 79% to 93%). SIRS criteria >1 was the most sensitive (94.3%, 95% CI 85.6% to 98.4%) but least specific (20.7%, 95%CI 13.4% to 30%). The PPV of SOFA >4 (68.3%, 95% CI 51.9% to 81.9%) was the highest in scoring system. The NPV of SIRS >1 (86%, 95% CI 73% to 94%) was similar to PSI >3 (86%, 95% CI 73% to 94%) and higher than the other score.

In terms of the secondary outcome, discrimination of ICU admission using SIRS criteria was significantly smaller compared to CURB-65, PSI, qSOFA, SOFA, and APACHE II (P = 0.001, P = 0.005, P = 0.003, P = 0.006, and P = 0.008, respectively). On the other hand, there was no significant difference in multiple pairwise comparisons between CURB-65 and PSI (P = 0.65), CURB-65 and qSOFA (P = 0.52), CURB-65 and SOFA (P = 0.64), CURB-65 and APACHE II (P = 0.46), PSI and qSOFA (P = 0.76), PSI and SOFA (P = 0.94), PSI and APACHE II (P = 0.70), qSOFA and SOFA (P = 0.79), qSOFA and APACHE II (P = 0.96), and SOFA and APACHE II (P = 0.78). 

## 4. Discussion

### 4.1. Etiologies

At least one respiratory virus was detected in 38.5% of the tested samples in the present study. This rate varies greatly in the studies conducted in different countries. In a multinational study, Sakr et al. reported that at least one virus was present in 7.7% of the patients admitted to the ICU due to SARI [7]. This rate was reported to be higher in China as 94.2% [8] and in Alaska as 90% [9]. The rate of influenza virus-positive cases in the present study was 28.3%. This rate was reported as 9% in a study from Jordan [10] and as 8% in a study from Kenya [11]. Higher rates of influenza virus-positive cases were reported in the Southwestern United States (52%) [12]. In the present study, H1N1pdm09 was detected in 54% of influenza-positive cases and in 15% of all patients with SARI. This rate was observed to be higher than those reported in most of the previous surveillance studies [9,11,13,14] and lower than those reported in the studies from Europe (24.9%) [15] and the Southwestern United States (52%) [12]. It is always difficult to compare the results of studies due to several reasons. The possible reasons include actual epidemiological differences, climate differences, methodological differences (such as study inclusion criteria, study period, patient age, diagnostic methods used, and tested virus panels), and failure in sample collection and recording. It is thought that the differences in the rates of influenza virus subtypes could be influenced by the study conducted during the seasonal influenza period.

In the present study, the mortality rate in the patients positive for influenza virus was 31.1%. This rate was much higher than those reported in similar studies in the literature [13,14]. However, similar to those reported in the literature [13,14], there was no significant difference between the patients with and without influenza virus in terms of mortality rate (P = 0.377).

Oseltamivir treatment [16] or early-onset treatment [17] has been reported to have a statistically significant effect on the mortality rate. In the present study, it was observed that treatment of oseltamivir or early antiviral treatment decreased the mortality although it was not statistically significant.

### 4.2. Severity scoring systems

The CURB-65 [18] and PSI [19] scores are extensively used to stratify severity of CAP patients and are recommended by guidelines [3], but debates on their performances are still ongoing. In parallel with the findings of the previous studies [20,21], the results of the present study also showed that the CURB-65 score could independently predict the mortality rate. On the other hand, McCartney et al. reported that CURB-65 might be misleading in young adults with some atypical pneumonia [22]. A metaanalysis of 23 prospective studies including patients with radiographically-confirmed CAP revealed that the PSI had the lowest false negative rate, meaning that the test was able to correctly identify patients who had nonsevere pneumonia and were at low risk of death. On the other hand, the CURB-65 was more specific in correctly classifying patients who had a greater likelihood of death [23]. In the present study, the PSI was more sensitive but less specific than CURB-65. Capelastegui et al. [20] reported the AUROC as 0.87 (95% CI, 0.84–0.89) for CURB-65 and 0.88 (95% CI, 0.84–0.89) for PSI in estimating the 30-day mortality. In a metaanalysis of 40 studies, Chalmers et al. [24] reported no significant difference between the AUC values of PSI and CURB-65 (P = 0.1). Similarly, there was also no significant difference in predicting mortality between these two tests in the present study (P = 0.890).

The Sepsis-3 consensus considered that the qSOFA criteria should be used to identify sepsis patients in ED [5]. In the study conducted by Freund et al. [25] and Seymour et al. [6], on patients with sepsis, the qSOFA was reported to have greater predictive validity as compared with the SOFA and SIRS criteria. In their study, Raith et al. [26] reported that the SOFA score had significantly greater discrimination for in-hospital mortality compared to the qSOFA and SIRS criteria. In the present study, there was no significant difference in comparisons between qSOFA and SOFA or between qSOFA and SIRS criteria, but there was a significant statistical difference only between SOFA and SIRS in predicting the 28-day mortality.

In the study, F. Tokioka et al. [27] reported that the predicting performance of qSOFA was lower than those of the CURB-65 (AUC = 0.687 vs. 0.773, P = 0.022) and PSI (AUC = 0.687 vs. 0.738, P = 0.036) for the 28-day mortality. In the present study, there was no significant difference among CURB-65, PSI, and qSOFA in multiple pairwise comparisons.

Desai and Lakhani [28] compared SOFA and APACHE II, in rural based ICU in patients having sepsis. They concluded that SOFA score was higher than APACHE II for predicting the outcome in sepsis patients. Kumar et al. [29] reported that the APACHE II and SOFA scores were significant for mortality in 168 confirmed or suspicious H1N1 critical patients, but there was also no significant difference in predicting mortality between these two tests. Similarly, there was no significant difference between SOFA and APACHE II in terms of predicting mortality comparisons in the present study.

Our study revealed that SIRS criteria > 1 was the most sensitive predictor of mortality. The metaanalysis of sensitivity for the diagnosis of sepsis comparing the qSOFA and SIRS was in favor of SIRS [30]. Given that a scoring system should have a high sensitivity to identify low-risk patients, the SIRS criteria seem to be more useful than the other score in the prehospital setting. But, application of SIRS criteria did not stratify severity for the SARI patients. When only SIRS criteria was applied, three in 159 patients were not diagnosed with sepsis.

In conclusion, SOFA and APACHE II were more accurate than SIRS in predicting the 28-day mortality among adults with severe acute respiratory infection admitted to ED. There was no significant difference in other multiple pairwise comparisons for mortality. Moreover, the results of the present study contributed to the underlying etiology of hospitalized SARI patients and emphasized the importance of influenza virus as a dominant virus associated with viral etiology.

This study has some limitations. Firstly, the present study was conducted in a single center; therefore, the results of the present study could not be generalized. Secondly, bronchoalveolar washing or sputum culture is not routinely performed for SARI patients in our ED; therefore, we could not comment on the effects of different causal pathologies on clinical outcomes. Finally, the effects of complex interactions such as body mass index, race, ethnicity, nutritional status, preinfluenza story, and socio-economic factors should also be kept in mind.
